# Upregulation of CYP 450s expression of immortalized hepatocyte-like cells derived from mesenchymal stem cells by enzyme inducers

**DOI:** 10.1186/1472-6750-11-89

**Published:** 2011-09-30

**Authors:** Khanit Sa-ngiamsuntorn, Adisak Wongkajornsilp, Kanda Kasetsinsombat, Sunisa Duangsa-ard, Lalana Nuntakarn, Suparerk Borwornpinyo, Pravit Akarasereenont, Somchai Limsrichamrern, Suradej Hongeng

**Affiliations:** 1 Department of Pharmacology, Faculty of Medicine Siriraj Hospital, Mahidol University, 2 Prannok Road, Bangkoknoi, Bangkok 10700, Thailand; 2 Department of Surgery, Faculty of Medicine Siriraj Hospital, Mahidol University, 2 Prannok Road, Bangkoknoi, Bangkok 10700, Thailand; 3 Department of Pediatrics, Faculty of Medicine Ramathibodi Hospital, Mahidol University, 270 Rama VI Road, Ratchatewi, Bangkok 10400, Thailand; 4 Department of Pathology, Faculty of Medicine Ramathibodi Hospital, Mahidol University, 270 Rama VI Road, Ratchatewi, Bangkok 10400, Thailand; 5 Department of Biotechnology, Faculty of Science, Mahidol University, 272 Rama VI Road, Ratchatewi, Bangkok 10400, Thailand

**Keywords:** hepatocyte-like cell, immortalization, CYP450, MSC

## Abstract

**Background:**

The strenuous procurement of cultured human hepatocytes and their short lives have constrained the cell culture model of cytochrome P450 (CYP450) induction, xenobiotic biotransformation, and hepatotoxicity. The development of continuous non-tumorous cell line steadily containing hepatocyte phenotypes would substitute the primary hepatocytes for these studies.

**Results:**

The hepatocyte-like cells have been developed from hTERT plus Bmi-1-immortalized human mesenchymal stem cells to substitute the primary hepatocytes. The hepatocyte-like cells had polygonal morphology and steadily produced albumin, glycogen, urea and UGT1A1 beyond 6 months while maintaining proliferative capacity. Although these hepatocyte-like cells had low basal expression of CYP450 isotypes, their expressions could be extensively up regulated to 80 folds upon the exposure to enzyme inducers. Their inducibility outperformed the classical HepG2 cells.

**Conclusion:**

The hepatocyte-like cells contained the markers of hepatocytes including CYP450 isotypes. The high inducibility of CYP450 transcripts could serve as a sensitive model for profiling xenobiotic-induced expression of CYP450.

## Background

Xenobiotic biotransformation has been classified into 2 phases. The majority of phase I biotransformation was implemented by cytochrome P450 (CYP450) family with 8 major isotypes in human[1]. Each isotype has overlapped spectra of substrates and catalyzes multiple reactions. Activations or suppressions of certain isotypes as a result of precipitant drugs have been associated with several clinically important drug interactions[1]. The phase II biotransformation involved several conjugation reactions (e.g., sulfonation, glucuronidation, acetylation, methylation and glutathione conjugation). These conjugations attach new functional groups to the xenobiotic that had gone through phase I metabolism[2]. The CYP450 isotypes in rodents are often different in gene regulation and enzymatic activity from those in human and thus cannot reliably predict the toxicity or metabolic profiles of xenobiotics in human[3].

The idealistic cell culture model to simulate *in vivo *biotransformation of xenobiotics is the use of primary human hepatocytes. However, the acquisition of normal human hepatocytes is cumbersome with ethical as well as biological considerations. The cultured cells are short lived and have to be swiftly prepared from fresh tissues[4] making them unfeasible for most studies. Alternative sources of human cells have been developed to mimic the phenotypes of hepatocytes. A viable source is the mesenchymal stem cells (MSCs) derived from bone marrow[5].

The very first effort to generate hepatocyte-like cells was taken through the co-culture of MSCs with isolated liver cells[6]. Subsequent efforts employed fetal liver-conditioned medium[7], selective cytokines and coating matrix[8]. Alternate cell sources such as adipose tissue[9-11], amniotic fluid[12] and Wharton's jelly[13-15] were employed. The major proposed application of these hepatocyte-like cells is to implement liver regeneration[13,16-19]. The xenogeneic transplants of human hepatocyte-like cells into mice after CCL_4_-induced liver injury have been attempted with moderate success[5,20,21]. Several groups had characterized the phenotypes (i.e., CYP450, morphology, glycogen/urea/albumin production) in contemporary hepatocyte-like cells[22], but none has made the long-term characterization to demonstrate their stability. The long-term stability of the cells is required for the application of xenobiotic testing in new drug development.

The life span of hepatocyte-like cells from these diverse sources after differentiation induction was generally limited. Immortalizing hepatocyte-like cells or their precursors (i.e., MSCs) would be a more feasible solution, resulting in a sustainable and consistent source of hepatocytes. The polycomb group transcription factor Bmi-1[23] that could drive cancer cell proliferation[24] and normal stem cell self-renewal was selected for immortalization. The validity for using these immortalized cells for cell culture metabolic study relies on the maintenance of hepatocyte phenotypes as represented by a panel of specific markers. Hepatocyte-like cells from various MSC sources exhibited different intensities of hepatocyte specific markers[9]. We immortalized the MSC as a precursor for hepatocyte-like cells by using both human telomerase reverse transcriptase gene (hTERT) and Bmi-1 through lentiviral transduction[25], and examined whether the resulting immortalized cells after differentiation induction could maintain hepatocyte phenotypes and metabolic functions.

## Results

### The identification of MSCs

**Figure 1:**
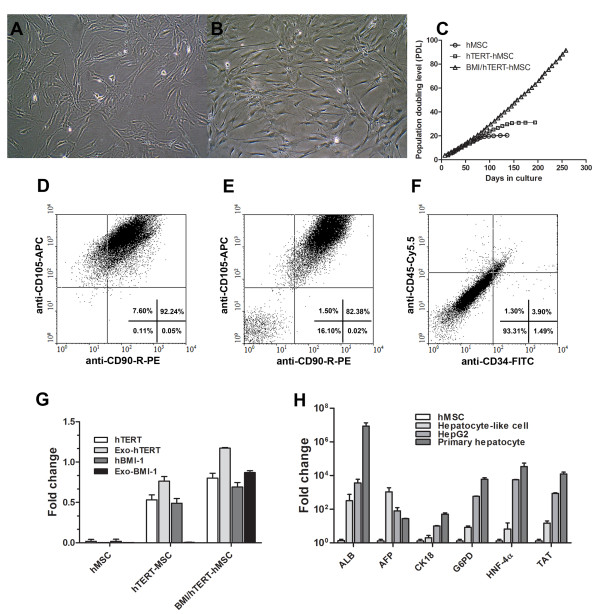
**Characterization of immortalized MSC**. MSCs and Bmi-1/hTERT-immortalized MSCs were visualized (A) after the 2^nd ^passage. The attached cells appeared fibroblast-like, spindle morphology (B) at the 40^th ^passage (12 months after isolation). The MSCs (hMSC) and the TERT-transduced MSCs (hTERT-MSC), or the double TERT/Bmi-1 transduced MSCs (BMI/hTERT-MSC) were studied for cumulative population doubling level (PDL) (C). Flow cytometry analysis confirmed the presence of CD90/CD105 in primary MSCs after isolation (D) and in BMI/hTERT-MSCs (E). All cells were depleted of CD35/CD45 hematopoietic stem cell markers (F). The endogenous and exogenous expression of Bmi-1, TERT in all cell types were studied using quantitative real-time PCR (G). The expression of hepatocyte-selective genes (i.e., albumin (ALB), α-fetoprotein (AFP), cytokeratin18 (CK18), glucose-6-phosphate dehydrogenase (G6PD), hepatocyte nuclear factor (HNF-4α), and tyrosine aminotransferase (TAT)) of BMI/hTERT-MSC after hepatic differentiation was presented as fold change over the untreated MSCs in comparison with HepG2 and the primary hepatocyte (H).

Cells isolated from bone marrow aspirate displayed a spindle shape upon reaching confluence (Figure 1A). The hTERT/Bmi-1-transduced MSC (BMI1/hTERT-MSC) still maintained fibroblast-like, spindle morphology at 40^th ^passage (Figure 1B) with an exponential growth pattern (Figure 1C). The identity of the studied MSCs was confirmed by the presence of mesenchymal stem cell markers (CD90 and CD105, Figure 1D). MSCs that had gone through immortalization still contained similar levels of CD90 and CD105 (Figure 1E), but was virtually devoid of hematopoietic markers (CD34, CD45, Figure 1F) as determined by a flow cytometer.

### Proliferative activity of transformed MSCs

The growth rate of MSCs was slow at the first passage, picked up and steadily increased in subsequent (2^nd^-7^th^) passages. In later (8^th ^- 10^th^) passages, growth rate was again slowed down to a complete stop (Figure 1C). To bypass the replication senescence, we transformed MSCs with either hTERT plus Bmi-1 (BMI/hTERT-MSC) or hTERT alone (hTERT-MSC) from 5 independent donors. After 60 days or 20-25 population doubling level (PDL), the proliferation rate of untreated MSCs decreased to a final stop. The cellular morphology switched to epithelial-like, indicating the reduction of stem cell properties. In contrast, BMI/hTERT-MSC grew steadily for more than 8 months (75-80 PDL) and exhibited unaltered morphology (Figure 1C). In contrast, hTERT-MSCs, similar to untreated MSCs, could not bypass replication senescence. BMI/hTERT-MSC cells have been maintained for over a year, confirming their immortalization. To ensure that both Bmi-1 and hTERT were expressed in these transformed MSCs, specific primers were designed to separately quantify endogenous and exogenous expression of both Bmi-1 and hTERT using quantitative RT-PCR. The endogenous expressions of hTERT and hBmi-1 in untreated MSCs at the 4^th ^passage were lower than those in transformed MSCs at the same passage. The ectopic expressions of both hTERT and Bmi-1 were detected at a steadily high level for over a year (120 PDL, Figure 1G). The morphology was also stable throughout the study. Our success rate for immortalization of MSC was 4 clones out of 10 clones from each donor.

### The differentiation of MSCs to hepatocyte-like cells

**Figure 2:**
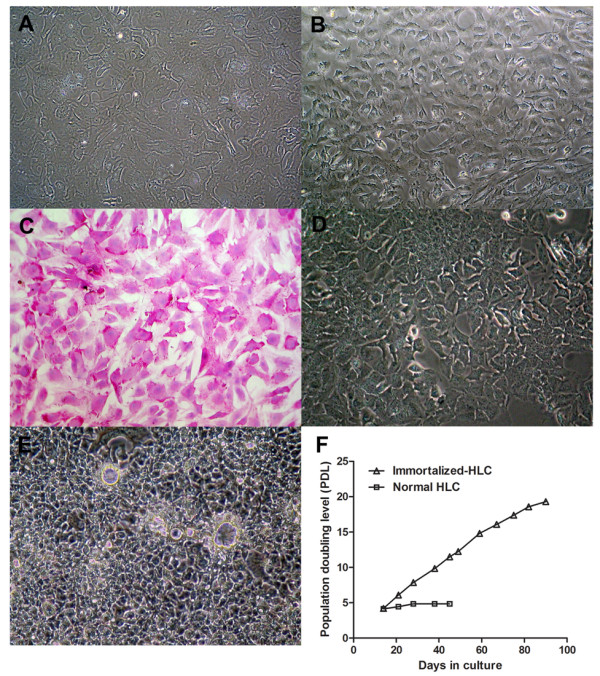
**Characterization of immortalized MSC-derived hepatocyte-like cells**. The TERT/Bmi-1-transduced MSCs had been differentiated into hepatocyte-like cells. The differentiated cell had polygonal shape, granulated cytoplasm and large nucleus (A). After the next passage, the cytoplasmic/nucleolus ratio was further decreased with loose intercellular attachment (B). The glycogen storage activity was demonstrated using Periodic Acid Schiff staining (PAS) with greater than 95% of the population were positive for glycogen (C). After the maintenance in DMEM/F12, 10% FBS in subsequent passages, cells were densely packed with closer intercellular attachment (D). After reaching confluence, cells formed duct-like structure (E). The life span of the immortalized hepatocyte-like cells was beyond 3 months (F) with active division.

After finishing hepatic induction, the hepatocyte-like cells carried the expansion of several basic hepatocyte genes (Figure 1H) with a corresponding polygonal morphology (Figure 2A). Immortalized hepatocyte-like cells at the first passage were loosely attached to adjacent cells (Figure 2B). Up to 70-80% of the hepatocyte-like cells deposited glycogen, especially in densely populated area (PAS assay at passage 4, Figure 2C). After switching to 10% FBS, DMEM/F12 the intercellular attachment was denser with blurring of cell boundary (Figure 2D). At confluence, duct-like structure was observed (Figure 2E). The cells could maintain cell division beyond 3 months (Figure 2F) with sustainable hepatocyte function suitable for drug screening.

### Expression levels of hepatocyte-specific markers

**Figure 3:**
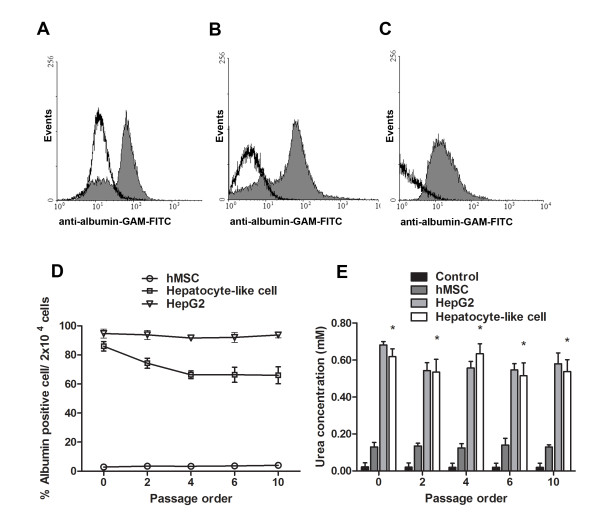
**Functional activities of hepatocyte-like cells**. The presence of albumin was analyzed using flow cytometer in the immediately differentiated cells (A), the immortalized hepatocyte-like cells (B), and in HepG2 (C). Greater than 80% of the population of hepatocyte-like cells contained albumin (D) that could be maintained at 70% beyond 10 passages. Urea production as represented by diacetyl monoxime test was demonstrated in MSCs, HepG2, and hepatocyte-like cells (E). IMDM medium supplemented with 5 mM NH_4_Cl served as the control group.

Up to 88% of hepatocyte-like cells (Figure 3A) and immortalized hepatocyte-like cells (Figure 3B), as opposed to 5% of MSCs, contained intracellular albumin. Almost 95% of HepG2 cells contained albumin (Figure 3C). In subsequent passages, hepatocyte-like cells maintained at least 70% of albumin-containing cells (Figure 3D). The functional activity of hepatocyte-like cells at different passages was investigated using urea assay (Figure 3E). The conditioned medium of hepatocyte-like cells contained far higher level of urea than that of MSCs, but was comparable to that of HepG2. Using mRNA levels inherent to MSCs as a reference, the relative expression levels of the corresponding genes in the hepatocyte-like cells, HepG2 cells and the primary human hepatocytes were determined using quantitative real-time PCR. The basal expression patterns for hepatocyte-specific genes at passage 5-9 (e.g., ALB, AFP, CK18, G6PD, HNF-4α and TAT) were varied, depending on the stage of hepatocyte maturation. The AFP expression that is usually presented in hepatic progenitors was detected at higher level than those of the primary human hepatocytes and HepG2 cells (Figure 1H). The observation of cytokeratin18 expression confirmed the differentiation of MSCs into endodermal tissue. Three major hepatocyte genes were up-regulated in hepatocyte-like cells, namely glucose-6-phosphate dehydrogenase (glucose metabolism), HNF-4α (liver development and maturation) and tyrosine aminotransferase (amino acid metabolism). The observation of all hepatocyte specific genes confirmed that the transformed MSCs could actually differentiate into functional hepatocyte-like cells.

### The basal expression of phase I and phase II enzymes in hepatocyte-like cell

**Figure 4:**
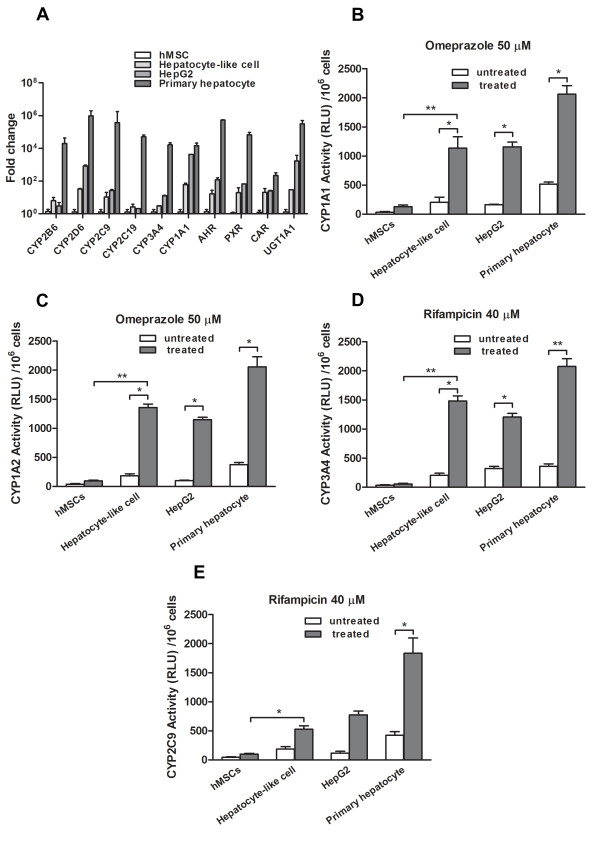
**Basal CYP450 activity and its inducibility**. The basal expression of CYP450 isotypes, CYP450 nuclear transcription factors, and UGT1A1 in MSCs, hepatocyte-like cells, HepG2 and the primary hepatocyte were analyzed using real-time qPCR (A). They were analyzed as fold-changes over that of untreated MSCs. The induction of CYP1A1 (B), CYP1A2 (C), CYP3A4 (D) or CYP2C9 (E) activities after adding the corresponding enzyme inducers for 72 h were analyzed using P450Glo™ assay kit (Promega) with different luciferin substrates. After 3-h incubation with specific substrate, luciferase activities were measured. Results are expressed as luciferase activities in relative luminescence unit (RLU) and mean ± SD of 3 independent experiments.

After maturation induction, hepatocyte-like cell culture was continued in IMDM with 1% FBS, 2% DMSO for 2 weeks at confluence [26]. Cells were harvested and determined for phase I and phase II enzyme expressions (Figure 4A). Specific transcription factors such as AHR, PXR and CAR in hepatocyte-like cells were increased by about 10 times those of MSCs. These genes are involved in the transcription of CYP450 isotypes [27,28]. Genes that were highly expressed in hepatocyte-like cells included one phase II enzyme UGT1A1 and 6 CYP450 isotypes (CYP2B6, CYP2D6, CYP2C9, CYP2C19, CYP3A4, and CYP1A1). In particular, the expression level of CYP2B6 in hepatocyte-like cells was even higher than that of HepG2 while other isotypes achieved comparable expression levels to those of HepG2. However, all CYP450 isotype expressions in hepatocyte-like cells were only 10-20% that of normal hepatocytes. The authenticity of the real-time RT-PCR products of hepatocyte markers and CYP450 were confirmed through the analysis for melting curve using Sequence Detection Software version 2.01 (Applied Biosystems, CA).

### Enhancing phase I enzyme expression using prototypic inducers

The expressions of CYP1A1, CYP2B6, CYP2D6 and CYP2C8 in hepatocyte-like cells were significantly increased to 13, 30, 3, 12 folds respectively after the induction with dexamethasone or rifampicin (Table 1). CYP3A4 and CYP2C19 expressions were extensively up regulated by 84 and 20 folds, respectively in hepatocyte-like cells. In HepG2, the expressions of CYP2B6, CYP2C8 and CYP3A4 were increased to 25, 9, 39 folds using rifampicin. In the primary hepatocyte, the expression of CYP1A1, CYP1A2, CYP2B6 were raised to 19, 13, 41 folds that were comparable to those of the hepatocyte-like cells. The induction of CYP3A4 expression in the hepatocyte-like cells (84 folds) outpaced that of the primary hepatocyte (72 folds). The expressions of most CYP450 isotypes in undifferentiated MSCs were induced by merely 2-4 folds.

**Table 1:** Fold changes of CYP450 isotypes' expression in three cell types after 72 h of induction with prototypic CYP450 inducers (omeprazole, dexamethasone, rifampicin, artemisinine and ethanol) over the untreated control

P450 isotypes/enzyme inducers	concentration (μM)	mRNA fold change (mean ± SD)			
		
		HepG2	P-hepatocyte	hMSC	Hep-like cell
**CYP1A1**					
omeprazole	50	4.05 ± 0.38*	11.13 ± 0.34*	2.09 ± 0.30	2.13 ± 0.15
dexamethasone	25	1.11 ± 0.13	19.68 ± 0.46*	3.10 ± 0.33*	12.86 ± 0.39*
**CYP1A2**					
omeprazole	50	3.33 ± 0.14*	6.44 ± 0.80	1.67 ± 0.12	2.37 ± 0.13
dexamethasone	25	1.17 ± 0.02	13.62 ± 2.84*	3.82 ± 0.96*	5.63 ± 0.65*
**CYP2B6**					
rifampicin	40	25.82 ± 1.30*	35.16 ± 2.76**	1.46 ± 0.10	30.70 ± 5.36**
dexamethasone	25	2.58 ± 0.72	41.49 ± 4.58**	3.98 ± 0.71*	24.25 ± 2.30*
**CYP2D6**					
dexamethasone	25	1.26 ± 0.10	5.70 ± 1.29	2.68 ± 0.43	2.90 ± 0.73
**CYP2C9**					
rifampicin	40	1.42 ± 0.25	15.25 ± 2.51*	2.03 ± 0.22	7.78 ± 1.82*
**CYP2C19**					
rifampicin	40	1.87 ± 0.11	29.05 ± 2.02**	2.42 ± 0.61	19.01 ± 2.51*
**CYP2C8**					
rifampicin	40	9.07 ± 1.03*	24.40 ± 2.48*	3.03 ± 0.62*	12.28 ± 0.81*
**CYP3A4**					
rifampicin	40	39.43 ± 5.53**	69.50 ± 6.84**	3.64 ± 1.82*	43.29 ± 3.27**
dexamethasone	25	23.18 ± 7.25**	72.27 ± 5.64**	2.87 ± 0.96	84.10 ± 9.25**
artemisinine	50	48.18 ± 6.80**	59.28 ± 8.41**	0.89 ± 0.07	53.54 ± 1.37**
**CYP2E1**					
ethanol	88	4.59 ± 0.53*	24.23 ± 0.41*	1.46 ± 0.19	10.74 ± 2.10*

hMSC, HepG2, primary hepatocyte and hepatocyte-like cell were induced with prototypic inducer drugs. The CYP450 mRNA level was detected by Real-time PCR. The experiment was performed in triplicate for all compounds. Data was normalized by untreated (non-treated controls) and was analyzed by Non parametric One-tailed Student t test (Mann-Whitney test) * p < 0.05, ** p < 0.01.

### Substantial induction of CYP1A1, CYP1A2, CYP2C9 and CYP3A4 isotype activities in hepatocyte-like cells

**Figure 5:**
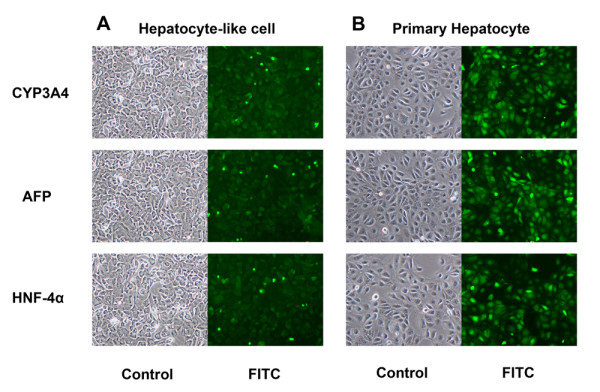
**Immunofluorescent staining of hepatocyte-like cells and the primary hepatocyte**. The immunofluorescent staining of CYP3A4, α-fetoprotein and HNF-4α in the cytoplasm of hepatocyte-like cells (A) and the primary hepatocyte (B) were demonstrated with the corresponding phase-contrast pictures over the same fields (10× objective lens). Essentially all hepatocyte-like cells carried all 3 proteins but their staining intensities reached only a half those of the primary hepatocytes.

Using luminescent CYP450-specific substrates, we determined CYP1A1, CYP1A2, CYP2C9 and CYP3A4 isotype activities after the induction with either rifampicin or omeprazole in HepG2, MSC and hepatocyte-like cell. We observed end-point catalytic activity after incubating substrates to the cells using a luminometer. In hepatocyte-like cell, the activity of CYP1A1, CYP1A2 and CYP3A4 was increased to approximately 6-7 folds that of the untreated cell (Figure 4B, C, D). A mild increase in CYP2C9 activity (2 folds) was observed (Figure 4E). The activity of CYP1A2 and CYP3A4 in hepatocyte-like cells was already higher than those in HepG2. The primary hepatocyte provided the highest activities in all CYP450 isotypes. A significant increase in rifampicin-induced CYP3A4 activity was confirmed by the accumulation of CYP450 in hepatocyte-like cell as demonstrated by immunofluorescent staining (Figure 5A). The staining for AFP and hepatocyte nuclear factor 4α confirmed the identity of the hepatocyte-like cell. The corresponding staining to the primary hepatocyte served as the positive controls (Figure 5B). No significant induction was detected in untreated MSCs, but untreated HepG2 and untreated hepatocyte-like cell had low basal level of CYP1A1, CYP1A2, CYP2C9 and CYP3A4 activities.

## Discussion

The hepatocyte-like cells have been developed to replace the primary hepatocytes for the studies of xenobiotic-induced CYP450 isotype expression, hepatotoxicity, and xenobiotic biotransformation. Taken together, the cell morphology, cell-selective markers (i.e., gene expression profiles, flow cytometry) as well as certain specific phenotypes (urea synthesis, glycogen deposit, and CYP450 expression profiles) indicated that the putative hepatocyte-like cells were correctly driven toward hepatocyte differentiation. To our knowledge, there has not been any attempt to bring hepatocyte-like cells derived from MSC as a stable model for the study of CYP450 isotypes or new drug development. Only certain isotypes (CYP1A1, CYP1A2, CYP2B6, CYP7A1 and CYP2E1) have been studied immediately after differentiation [18,29,30]. The expression of various transcription factors that regulate CYP450 isotypes[31] including hepatocyte nuclear factor (HNF-4α) [32] during hepatogenic differentiation has been reported. PXR, AHR and CAR are considered to be the most important regulators of xenobiotic-induced regulation of many CYP450 isotypes[28]. We observed increasing expression of PXR, CAR and HNF-4α in correlation with the degree of hepatocyte-like cell maturation.

Several investigators had developed hepatocyte-like cells from different stem cell sources and various differentiation protocols[33]. The MSC sources were bone marrow, adipose tissue and Wharton's jelly[10,29,34,35]. The differentiation phenotypes were generally lost after a few days in all studies including one employing embryonic stem cells as precursors[36]. The senescence of the precursor MSCs led to decreasing both proliferation and plasticity [37-39]. Our MSCs also reached senescence after 20-24 population doublings, similar to others [40-42]. The generation of hepatocyte-like cells from MSCs has been plagued by the lack of stable supply of the precursor MSCs and their differentiation capacity. These obstacles would be obviated if immortalized hepatocyte-like cells with intact phenotypes could be generated.

A classical gene employed for immortalization is the telomerase reverse transcriptase (hTERT) that prevents replicative senescence associated with decreasing telomere length resulting from repeated cell division [43,44]. Lesser known immortalization genes are SV-40 large T antigen (T-Ag) and Bmi-1[25]. Bmi-1 inhibit senescence and extended the life span of normal human cell by suppressing p16^INK4A ^that allows cell entry into division[45]. Moreover, the overexpression of Bmi-1 could inhibit TGF-β signaling[46] that would otherwise induce hepatocyte cell death[47].

MSCs lost their originally high telomerase activity [48] after being seeded as primary cultured cells [49] and eventually lost their stem cell property [50]. We had expected that the addition of a constitutively expressing construct of hTERT (pLOX-TERT-iresTK) to MSCs would solve the senescence [51,52], but a slight postponement of senescence was observed. Only after Bmi-1 (pLOX-CWBmi1)[53] had been co-introduced were the senescence phenotypes extensively delayed. The transduced cells had increasing spindle morphology with at least 80% of the whole population contained both CD90 and CD105. These cells could be maintained for over 1 year while still maintaining their growth in exponential phase. The given constructs have an advantage of the inclusion of *loxP *site that allows the removal from the genomic insertion site.

It is even more exciting when the precursor cells of the hepatocyte-like cells were already immortalized. We modified the hepatogenic differentiation protocol from previous reports [54] by extending the last step of maturation from 2 weeks to 4 weeks in culture medium supplemented with 2% DMSO. Normally, we cannot achieve a complete hepatocyte differentiation. However, after using limiting dilution technique, a stable clone of functional hepatocyte-like cell was established. Our clone of differentiated immortalized cells could propagate in standard culture condition for greater than 6 months with sustainable hepatocyte specific makers and functions.

The confirmed hepatocyte phenotypes that included the expression of albumin, α-fetoprotein cytokeratin 18, HNF-4α, and tyrosine aminotransferase were up-regulated for 10 - 100 folds that of the undifferentiated MSCs. However, the overall basal gene expressions were 10 - 40% those of the primary hepatocyte. The flow cytometry analysis indicated that at least 80% of the hepatocyte-like cells, in comparison with 90% of HepG2, produced albumin. Likewise, the urea production of our cells was comparable to that of HepG2. Greater proportion of our hepatocyte-like cells (> 95%) carried glycogen than did others[9,30,55,56]. The expression of transcription factors for CYP450 (i.e., CAR, AHR, PXR) as well as that of the phase II enzyme (i.e., UGT1A1) was observed. The 10 - 50-fold induction of the expression of 8 major CYP450 isotypes (CYP1A1 CYP1A2, CYP2B6, CYP2D6, CYP2C9, CYP2C19, CYP2C8, CYP3A4 and CYP2E1) in response to known enzyme inducers (rifampicin, dexamethasone, omeprazole, phenobarbital and artesunate) was confirmed, although their basal levels were less than those of the primary hepatocyte by 100-1000 folds. Unexpectedly, HepG2 achieved much weaker induction of CYP1A1, CYP2D6, CYP2C9 and CYP2C19 in response to rifampicin, dexamethasone and omeprazole than did hepatocyte-like cells. The immunofluorescent study of CYP3A4 in hepatocyte-like cells after induction indicated that the up-regulation of protein level was consistent with the induction of mRNA expression.

Although CYP450 isotypes are presented in most cell types, not all cell types are suitably employed to study the CYP450's response to xenobiotics. To elucidate the suitability of the hepatocyte-like cells for the study of CYP450 isotypes, we have extensively investigated the expressions of all major isotypes plus the enzymatic activity of selected isotypes in response to enzyme inducers. We have demonstrated that the un-modified MSCs contained low basal levels of CYP450 isotypes and elicited only 2 - 5-fold induction to prototypic CYP450 isotype inducers (Table 1). Therefore, the use of MSCs is not considered a viable alternative for CYP450 study. We observed extensively high expressions of most CYP450 isotypes in response to inducers in hepatocyte-like cells than those in MSCs or HepG2, although the basal levels of certain CYP450 isotypes were lower than those of primary hepatocytes or HepG2. Changing the precursors of hepatocyte-like cells from MSCs to embryonic stem cells or induced pleuripotential stem cells could not bring up the basal levels of all isotypes [57]. An exception was found in CYP2B6, where the hepatocyte-like cells had comparable expansion to that of the HepG2. The low basal levels of these CYP450 isotypes in hepatocyte-like cells might be attributed to their completely lack of exposure to xenobiotics as opposed to the primary hepatocytes or HepG2. Based on the expansion of CYP450 isotypes' expression in response to inducers, hepatocyte-like cells are considered a more sensitive and informative model.

## Conclusion

The continuous hepatocyte-like cell lines have been generated from hTERT plus Bmi-1-immortalized human MSCs. These continuous cell lines contained hepatocyte markers (albumin, AFP, TAT, HNF-4α, G6PD) including all major CYP450 isotypes (CYP1A1, CYP1A2, CYP2C8, CYP2B6, CYP2D6, CYP2C9, CYP2C19, CYP3A4 and CYP2E1). The basal mRNA expression of CYP450 isotypes was low, but readily up-regulated up to 80 folds upon the exposure to enzyme inducers. The high inducibility of CYP450 transcripts would serve as a sensitive model for profiling xenobiotic-induced expression of CYP450.

## Methods

### The characterization of human mesenchymal stem cells

Human mesenchymal cells (MSCs) were prepared from aspirated bone marrow of consenting normal volunteers (n = 5). This study received an approval from the Ethics Committee on Research Involving Human Subjects at Ramathibodi Hospital, Mahidol University. Written inform consent was obtained from all participants involved in this study. Bone marrow mononuclear cells were separated by IsoPrep (Robbins Scientific, Canada) density gradient centrifugation [58] and seeded at a density of 2 × 10^6 ^cells/mL in Minimum Essential Medium (MEM) α Media (Gibco Invitrogen, NY), 10% fetal bovine serum (FBS, Biochrom AG, Germany), 100 units/mL penicillin, 100 μg/mL streptomycin at 37°C in 5% CO_2_. The identification of MSCs was confirmed using FACS analysis. Isolated cells were detached by trypsin, stained for MSC markers (CD105 and CD90) or hematopoietic stem cell markers (CD34, CD45), and analyzed by flow cytometry (FACSCalibur, Becton Dickinson).

### Lentivirus production and transduction of target cells

Viral particles were produced using the transient transfection protocol[25]. HEK 293T cells (Clontech, CA) at a density of 2.8 × 10^6 ^cells/10-cm tissue culture dish were co-transfected with psPAX2 packaging vector, pMD2.G vesicular stomatitis virus G envelope, and the plasmid encoding either hTERT (pLOX-TERT-iresTK, Addgene plasmid 12245) or Bmi-1 (pLOX-CWBmi1, Addgene plasmid 12240)[53] using calcium phosphate precipitation. The supernatant was harvested and filtered through a 0.45 μm syringe filter. Viral stocks were stored at -70°C. For immortalization, both hTERT and Bmi-1 lentiviruses were diluted in MEM α medium, 10% FBS, 6 μg/mL polybrene at a multiplicity of infection (MOI) of 2, and directly added to the MSCs on six-well plates (10^4 ^cells/well). The MSCs were incubated at 5% CO_2_, 37°C for 14 h. After the incubation, medium containing viral particles was removed and replaced with fresh medium.

### Cloning of immortalized human mesenchymal stem cell

Three days after the infection, MSCs from 5 donors were trypsinized and counted using a hemacytometer. Single cell suspension was prepared by limiting dilution and transferred onto 24-well culture plate to establish clones from single cells. Each colony was monitored every 2-3 d until confluence. The cells were then trypsinized and seeded on T-25 tissue culture flask. To establish stable MSC lines, 10 clones per donor were selected based on the fastest cellular proliferation and confirmed for the expression of both hTERT and Bmi-1. Total RNA of MSC was isolated from pooled cells of passages 3-5, converted into cDNA and quantitated using real-time PCR. hTERT and Bmi-1 double positive cells were studied for population doubling level (PDL). The population doubling level was determined using log N/log2, where N is the number of the cells harvested at confluence divided by the number of the initially seeded cells [25].

### The induction of MSC hepatogenesis

The MSCs at passages 3-5 or BMI/hTERT-MSCs at a density of 1 × 10^4 ^cells/cm^2 ^from the fastest dividing clone (n = 3) were taken for differentiation. The MSCs were induced into hepatocyte-like cells using a modified three-step protocol[9]. They were maintained on collagen type IV coated container. The cells were maintained for 2 d in serum-free IMDM (Gibco Invitrogen, NY), 20 ng/mL epidermal growth factor (EGF, Chemicon, CA),10 ng/mL basic fibroblast growth factor (bFGF, Chemicon, CA). Cells were then maintained in IMDM (20 ng/mL HGF, Chemicon, CA), 10 ng/mL bFGF, and 0.61 g/L nicotinamide (Sigma, MO) for 7 d. Cells were further maintained in IMDM, 20 mg/mL oncostatin M (OSM, Chemicon, CA), 1 μM dexamethasone (Sigma, MO), and 50 mg/mL ITS^+ ^(Gibco Invitrogen, NY) for 14 d. The hepatogenesis was assessed by real-time PCR for liver-associated genes. Both human hepatocellular carcinoma cell line (HepG2) and the primary human hepatocyte served as controls. HepG2 was maintained in DMEM/F12 (Gibco Invitrogen, NY), 10% FBS, 100 units/mL penicillin (Sigma, MO), and 100 μg/mL streptomycin (Sigma, MO) at 37°C in 5% CO_2. _The primary human hepatocyte was maintained in Williams media E (Gibco Invitrogen, NY), 10% FBS, 100 units/mL penicillin (Sigma, MO), 100 μg/mL streptomycin (Sigma, MO), 4 μg/mL insulin and 1 μM dexamethasone (Sigma, MO) at 37°C in 5% CO_2._

### Urea production assay

MSCs, hepatocyte-like cells at passages 0-10 and HepG2 (HB-8065, ATCC, USA) were stimulated with 5 mM NH_4_Cl (Sigma, MO) for 48 h. The culture medium was collected and assayed for urea using diacetyl monoxime test[59]. The resulting diazine was measured at 540 nm with the SpectraMax M5 spectrofluorometer (Molecular Devices, CA).

### Glycogen Synthesis (Periodic Acid-Schiff, PAS) Assay

Immortalized hepatocyte-like cells at passage 4 were cultured on a chambered slide (Lab-Tek, Nunc, Denmark) for 3d. The slides were fixed in 4% formaldehyde, permeabilized with 0.1% Triton X-100 for 10 min, incubated with or without diastase for 1 h at 37°C, oxidized in 1% periodic acid (Sigma, MO) for 5 min, rinsed thrice with dH_2_O, treated with PAS reagent (Sigma, MO) for 15 min, and rinsed with water for 5 - 10 min. Samples were counterstained with Mayer's hematoxylin for 1 min, rinsed with water, and assessed under light microscope. The resulting gradient of oxidized glycogen would yield a gradient of color starting from pink to strong red.

### Analysis of cellular markers using flow cytometry

The cultured cells were stained with fluorochrome-conjugated to primary monoclonal antibodies raised against MSC markers (CD90, CD105); hematopoietic markers (CD34, CD45) (Biosource Invitrogen, CA). For intracellular albumin accumulation, hepatocyte-like cells at passages 2-10 were incubated with FACS Perm (BD Bioscience, CA) and stained with anti-human albumin (Abcam, MA). The goat anti-mouse IgG conjugated to FITC (Santa Cruz Biotechnology, CA) was used as the secondary antibody as necessary. The labeled cells were quantitated using a FACSCalibur flow cytometer (BD Bioscience). The data were analyzed using WinMDI version 2.9.

### Immunofluorescence Microscopy

Hepatocyte-like cells and the primary hepatocytes on chambered slide were washed twice with PBS, fixed with 4% paraformaldehyde for 30 min at room temperature followed by 100% ethanol for 10 min. The fixed cells were washed thrice with PBS, blocked with 5% normal serum from the same species as the secondary antibody in 1% BSA/0.2% Triton X-100/PBS for 1 h at room temperature. The cells were incubated with the primary antibody (anti-CYP3A4, anti-HNF4 or anti-α1-fetoprotein; Abcam, MA) for 1 h at 37°C, washed thrice, incubated with the secondary antibody for 1 h at 37°C, washed thrice, mounted with anti-fade mounting medium on coverslip, and examined under a fluorescent microscope.

### The induction of major CYP450 isotypes in hepatocyte-like cells using selective enzyme inducers

The modulation of expression levels of CYP450 isotypes was studied after the exposure to the classical inducers [1,22,60,61]. HepG2, MSC or hepatocyte like-cell from passages 3-7 at sub-confluent density were seeded on 6 well-plates for 48 h. These cells were treated for 72 h with the following agents: 40 μM rifampicin, 25 μM dexamethasone, 50 μM omeprazole, 1 mM phenobarbital, 50 μM artesunate, 88 μM ethanol or 0.1% (v/v) DMSO. The cell pellets were washed with 2-3 mL PBS, detached using 0.025% trypsin-EDTA, and neutralized with 10% FBS in IMDM. The cell pellets from passages 3-7 were pooled and stored at -80°C until analysis for CYP450 gene expression.

### Cell isolation, extraction of total mRNA and production of cDNA from primary hepatocyte, hepatocyte-like cell, MSCs and HepG2

The primary human hepatocytes were prepared from discarded surgical specimens using the 2-step collagenase method. The isolated cells were seeded over the collagen type IV-coated container and maintained in the above growth medium for 3 days. Total RNA isolation was performed using RNeasy Mini kit (Qiagen, Germany) according to the manufacturer's instruction. The quality and quantity of the total RNA were determined using a NanoVue Spectrophotometer (GE Healthcare, Buckinghamshire, UK). For cDNA synthesis, 2 μg of total isolated RNA from primary hepatocyte, hepatocyte-like cell, HepG2 and hMSC were converted to cDNA using the ImProm-II reverse transcription system (Promega, WI). Briefly, isolated RNA was incubated with 0.5 μg oligo (dT)_15 _primer in a total volume of 5 μL at 70°C for 5 min and chilled on ice-water immediately for at least 5 min. The reverse transcription mix (15 μL of 5X reaction buffer, 25 mM MgCl_2_, 2 mM dNTP Mix, 40 U/μL RNasin ribonuclease inhibitor, and 200 U/μL Improm-II^™ ^RT) was added to the RNA-primer mix to make a total volume of 20 μL. The mixture was incubated at 25°C for 5 min, and 42°C for another 1 h. The RT reaction was terminated by heating at 70°C for 15 min followed by chilling on ice. The cDNA samples were either used immediately or stored at -70°C. The 1.2 kb kanamycin RNA (1 μg) and non-template control served as positive and negative control system.

### Quantitative real-time PCR analysis for cell-specific markers

The employed hepatocyte markers included: ALB (albumin), AFP (α-fetoprotein), CK18 (cytokeratin18), G6PD (glucose-6-phosphate dehydrogenase), HNF-4α (hepatocyte nuclear factor 4α), and TAT (tyrosine amino transferase). The employed CYP450 markers included CYP1A1, CYP1A2, CYP2C8, CYP2B6, CYP2D6, CYP2C9, CYP2C19, CYP3A4 and CYP2E1. The primers for assessing P450s included those recognized aromatic hydrocarbon receptor (AHR), pregnane × receptor (PXR), constitutive aldosterone receptor (CAR). All gene specific primers were designed using Vector NTI version 10 (Invitrogen, Table 2) and ordered from 1st BASE (Singapore). They were amplified using FastStart SYBR^® ^Green Master (Roche diagnostic) and an ABI 7500 Sequence Detector (Applied Biosystems, CA) by following checklist information of RT-qPCR experiment (Additional file 1). Real-time PCR was performed using 5 μL of 10 μg/mL cDNA diluted in a 25 μL reaction mixture containing 0.4 μM for each primer and 12.5 μL SybrGreen with the following conditions: 95°C for 10 min, followed by 40 cycles of amplification at 95°C for 15 sec, 60°C for 40 sec, and 72°C for 40 sec. The fluorescent products were measured at the last step of each cycle. To determine the specificity of amplification, melting curve analysis was applied to all final PCR products, after finishing the thermal cycling. The non-template negative control (NTC) was performed with each gene-specific primer pair. The number of cycles required for the fluorescent signal to cross the threshold (Ct's) was determined from each primer pair. The obtained Ct's were subtracted with the Ct of the respective house-keeping gene (GAPDH) of the same cells to obtain ΔCt. To enable suitable comparison, the ΔCt's of the treated cells were subtracted with ΔCt's of the untreated cells of the same period to obtain ΔΔCt's. The relative fold change could be obtained from the expression of 2^-(ΔΔCt)^.

**Table 2:** Primer sets and conditions used in quantitative real-time PCR (qPCR)

Gene	Genbank Accession	Sense primer 5'----- > 3' (Tm°C)	Antisense primer 3'----- > 5' (Tm°C)	Size amplicon (bp)	Annealing temp. (°C)	Putative function
**ALB**	NM_000477	TGAGAAAACGCCAGTAAGTGAC (60.8)	TGCGAAATCATCCATAACAGC (58.7)	265	60	albumin

**AFP**	NM_001134	GCTTGGTGGTGGATGAAACA (60.4)	TCCTCTGTTATTTGTGGCTTTTG (59.2)	157	60	α-fetoprotein

**CK18**	X12881	GAGATCGAGGCTCTCAAGGA (62.4)	CAAGCTGGCCTTCAGATTTC (60.4)	357	60	cytokeration 18

**G6PD**	U01120	GCTGGAGTCCTGTCAGGCATTGC (68.1)	TAGAGCTGAGGCGGAATGGGAG (66.4)	349	60	glucose-6-phosphate dehydrogenase

**HNF-4α**	AY680696	GCCTACCTCAAAGCCATCAT (60.4)	GACCCTCCCAGCAGCATCTC (66.5)	256	60	hepatocyte nuclear factor 4α

**TAT**	NM_000353	TGAGCAGTCTGTCCACTGCCT (64.5)	ATGTGAATGAGGAGGATCTGAG (60.8)	338	60	tyrosine aminotransferase

**CYP2B6**	NM_000767	ATGGGGCACTGAAAAAGACTGA (60.8)	AGAGGCGGGGACACTGAATGAC (66.4)	283	60	Cytochrome P450 2B6

**CYP2D6**	NM_000106	CTAAGGGAACGACACTCATCAC (62.7)	GTCACCAGGAAAGCAAAGACAC (62.7)	289	60	Cytochrome P450 2D6

**CYP2C9**	NM_000771	CCTCTGGGGCATTATCCATC (62.4)	ATATTTGCACAGTGAAACATAGGA (57.7)	137	60	Cytochrome P450 2C9

**CYP2C19**	NM_000769	TTCATGCCTTTCTCAGCAGG (60.4)	ACAGATAGTGAAATTTGGAC (54.3)	277	60	Cytochrome P450 2C19

**CYP2C8**	NM_000770	ACAACAAGCACCACTCTGAGATATG	GTCTGCCAATTACATGATCAATCTCT	100	60	Cytochrome P450 2C8

**CYP3A4**	AK298451	GCCTGGTGCTCCTCTATCTA (62.4)	GGCTGTTGACCATCATAAAAGC (60.8)	187	60	Cytochrome P450 3A4

**CYP1A1**	BC023019	TCCAGAGACAACAGGTAAAACA (58.9)	AGGAAGGGCAGAGGAATGTGAT (62.7)	371	60	Cytochrome P450 1A1

**CYP1A2**	AF182274	ACCCCAGCTGCCCTACTTG (64.5)	GCGTTGTGTCCCTTGTTGT (62.4)	101	60	Cytochrome P450 1A2

**CYP2E1**	NM_000773	ACCTGCCCCATGAAGCAACC (64.5)	GAAACAACTCCATGCGAGCC (62.4)	246	60	Cytochrome P450 2E1

**PXR**	AB307701	GAAGTCGGAGGTCCCCAAA (62.3)	CTCCTGAAAAAGCCCTTGCA (60.4)	100	60	pregnane × receptor

**CAR**	AB307702	TGATCAGCTGCAAGAGGAGA (60.4)	AGGCCTAGCAACTTCGCACA (62.4)	102	60	constitutive androstane receptor

**AhR**	BC070080	ACATCACCTACGCCAGTCGC (64.5)	TCTATGCCGCTTGGAAGGAT (60.4)	101	60	aryl hydrocarbon receptor

**UGT1A1**	BC128414	GGAGCAAAAGGCGCCATGGC (62.5)	GTCCCCTCTGCTGCAGCTGC (64.5)	178	60	uridine diphosphate glucuronyltransferase 1A1

**LV-BMI-1**	NM_005180	GCTGAGGGCTATTGAGGCGCA (65.5)	ACCCCAAATCCCCAGGAGCTGT (65.7)	127	60	lentivirus vector BMI-1

**hBMI-1**	NM_005180	ACCTCCCAGCCCCGCAGAAT (65.9)	AGACGCCGCTGTCAATGGGC (66.4)	280	60	human BMI-1

**LV-hTERT**	AF018167	CAACCCGGCACTGCCCTCAG (66.9)	GGGGTTCCGCTGCCTGCAAA (68.2)	268	60	lentivirus vector telomerase reverse transcriptase

**hTERT**	AF018167	CGGAAGAGTGTCTGGAGCAAGT (59.5)	GAACAGTGCCTTCACCCTCGA (61.1)	258	60	human telomerase reverse transcriptase

Sequence of the primers and the conditions used in quantitative real-time PCR (qPCR).

### The analysis of CYP450 activity

CYP1A1, CYP1A2, CYP2C9 and CYP3A4 enzyme activities were assayed directly in all cultured cells (immortalized hepatocyte-like cell at passage 3-7, primary human hepatocyte, HepG2 or MSC) attaching to the collagen type IV-coated 6-well plate at a density of 10^6 ^cell/well. All cultured cells were divided into three groups. Group 1: cells were cultured in IMDM supplemented with 40 μM rifampicin to induce CYP450 isotypes 3A4 and 2C9. Group 2: cells were cultured in IMDM supplemented with 50 μM omeprazole for inducing CYP1A1 and CYP1A2. Group 3: cells were cultured in IMDM alone as a control. All conditions were incubated for 72 h with daily medium change. Metabolism was assessed based on luciferase activity using the P450-glo 1A1, 1A2, 2C9 and 3A4 assay (V8751, V8771, V8791, V9001; Promega, WI). After 3-d incubation period, cells were incubated with IMDM supplemented with 100 μM Luciferin-CEE, Luciferin-H, Luciferin-ME for 3-4 h or 3 μM Luciferin-IPA for 30-60 min. An aliquot (50 μL) of the medium was transferred to 96-well opaque white luminometer plate (Nunc, Denmark) and luciferin detection reagent was added to each well. After sitting at room temperature for 20 min, luminescence was measured with a SpectraMax M5 spectrofluorometer.

### Statistical analysis

Each experiment was performed in triplicate. Data were expressed as mean ± SD. Data from quantitative RT-qPCR and enzyme activity were evaluated for statistical significance using the Student's unpaired *t *test (p < 0.05). At least 3-fold induction in mRNA with statistically significant difference was judged as relevant.

## Authors' contributions

KS performed most of the experiments, designed the protocol, performed the statistical analysis and drafted the manuscript. AW, PA, and SH designed the protocol. SL participated in the preparation of the primary hepatocytes. LN and SB prepared the vectors. AW and SH coordinated the study. SD participated in MSC culture. KK participated in qRT-PCR. All authors have read and approved the final manuscript.

## Supplementary Material

Additional file 1 **Details of quantitative RT-PCR conditions**. This file contains the tabulated specific information for qRT-PCR section. Click here for file
