# Supplementary material for: Upregulation of CYP 450s expression of immortalized hepatocyte-like cells derived from mesenchymal stem cells by enzyme inducers
Source: BMC Biotechnol. 2011 Sep 30;11:89. doi: 10.1186/1472-6750-11-89 (PMC3198927; doi:10.1186/1472-6750-11-89)
Supplement: Additional file 1 — Details of quantitative RT-PCR conditions. This file contains the tabulated specific information for qRT-PCR section. [file 1472-6750-11-89-S1.DOC]

**Additional file 1.**

**Additional RT-qPCR methods: Key criteria of essential technical information required for the assessment of a RT-qPCR experiment.**

| **Sample/Template** | **Details** | **Checklist** |
| --- | --- | --- |
| Source | If cancer, was biopsy screened for adjacent normal tissue? |  |
| Method of preservation | Liquid N_2_/RNAlater/formalin | Liquid N_2_ |
| Storage time (if appropriate) | If using samples >6 months old | no sample store than 6 months |
| Handling | fresh/frozen/formalin | Fresh isolation (Cell culture) |
| Extraction method | TriZol/columns | Columns (Qiagen) |
| RNA: DNA-free | Intron-spanning primers/no RT control | No RT control |
| Concentration | Nanodrop/ribogreen/microfluidics | Nanodrop (NanoVue GE Health care,UK) |
| RNA: integrity | Microfluidics/3':5' assay | 3':5' assay |
| Inhibition-free | Method of testing |  |
| **Assay optimisation/validation** |  |  |
| Accession number | RefSeq XX_1234567 | see Table 2. |
| Amplicon details | exon location, amplicon size | see Table 2. |
| Primer sequence | even if previously published | see Table 2. |
| *Probe sequence** | identify LNA or other substitutions | no probe in this experiment |
| *In silico* | BLAST/Primer-BLAST/m-fold | BLAST |
| empirical | primer concentration/annealing temperature | 1µM |
| Priming conditions | oligo-dT/random/combination/target-specific | oligo-dT |
| PCR efficiency | dilution curve | dilution curve 10 fold-dilution |
| Linear dynamic range | spanning unknown targets | - |
| Limits of detection | LOD detection/accurte quantification | - |
| Intra-assay variation | copy numbers not Cq | - |
| **RT/PCR** |  |  |
| Protocols | detailed description, concentrations, volumes | see in methods |
| Reagents | supplier, Lot number | see in methods |
| Duplicate RT | DCq | Tripicate in ∆Cq |
| NTC | Cq & melt curves | Cq, melt cure and gel electrophoresis |
| NAC | DCq beginning:end of qPCR | ∆Cq 3:40 |
| Positive control | inter-run calibrators | Positive control in some gene (1.2 kb Kanamycin) |
| **Data analysis** |  |  |
| Specialist software | e.g., QBAsePlus | Sequence Detection Software (SDS v2.1) Applied Biosystems |
| Statistical justification | e.g., biological replicates | mean ± SD, Biological tripication |
| Transparent, validated normalisation | e.g., GeNorm summary | - |
